# Lower Oxytocin Plasma Levels in Borderline Patients with Unresolved Attachment Representations

**DOI:** 10.3389/fnhum.2016.00125

**Published:** 2016-03-30

**Authors:** Andrea Jobst, Frank Padberg, Maria-Christine Mauer, Tanja Daltrozzo, Christine Bauriedl-Schmidt, Lena Sabass, Nina Sarubin, Peter Falkai, Babette Renneberg, Peter Zill, Manuela Gander, Anna Buchheim

**Affiliations:** ^1^Department of Psychiatry and Psychotherapy, Ludwig Maximilian UniversityMunich, Germany; ^2^Department of Psychology, Freie Universität BerlinBerlin, Germany; ^3^Department of Clinical Psychology II, Institute of Psychology, University of InnsbruckInnsbruck, Austria

**Keywords:** oxytocin, attachment representation, borderline personality disorder, social exclusion, cyberball, social pain

## Abstract

Interpersonal problems and affective dysregulation are core characteristics of borderline personality disorder (BPD). BPD patients predominantly show unresolved attachment representations. The oxytocin (OT) system is associated with human social attachment and affiliative behavior, and OT dysregulation may be related to distinct attachment characteristics. Here, we investigated whether attachment representations are related to peripheral OT levels in BPD patients. Twenty-one female BPD patients and 20 age-, gender-, and education-matched healthy controls (HCs) were assessed with clinical scales and measures of interpersonal and attachment-related characteristics, including the Adult Attachment Projective Picture System (AAP). Plasma OT concentrations were measured prior to and during social exclusion in a virtual ball tossing game (Cyberball). The majority of BPD patients (63.2%) but no HCs showed unresolved (disorganized) attachment representations. In this subgroup of patients, baseline OT plasma levels were significantly lower than in BPD patients with organized attachment representations. This pilot study extends previous findings of altered OT regulation in BPD as a putative key mechanism underlying interpersonal dysregulation. Our results provide first evidence that altered OT plasma levels are related to disorganized attachment representations in BPD patients.

## Introduction

Borderline personality disorder is characterized by a pervasive pattern of emotional instability, impulsivity, severe problems in social interactions, and disturbed self-image (Bohus et al., 2009; Leichsenring et al., 2011). Particularly dysfunctional interpersonal beliefs and behavior, such as interpersonal events like rejection or exclusion, have a high clinical relevance and result in increasing aversive tension in BPD, which may lead to self-injurious behavior or dissociation (Herpertz, 1995; Stiglmayr et al., 2005; Gunderson and Lyons-Ruth, 2008). Interpersonal styles of BPD patients are characterized by a need for closeness and attention, combined with a fear of rejection and abandonment. Winter et al. (2015) found a negative evaluation bias for positive, self-referential information in BPD patients suggesting that they tend to refer information to themselves even if no explicit reference context is set. At the same time, BPD patients demonstrate significant impairments in making social judgments about others from their faces by judging them as less trustworthy and approachable than controls (Nicol et al., 2013). Moreover, Fertuck et al. (2009) reported an enhanced mental state discrimination based on the eye region of the face in BPD patients compared to HCs and considered this enhanced sensitivity as a potential basis for social impairment in BPD. Gunderson and Lyons-Ruth (2008) describe a genetically based hypersensitivity to interpersonal stressors in BPD patients: An interpersonal hypersensitivity phenotype may lead to characteristic interpersonal strategies and contradictory interpersonal features observed in adult BPD patients.

Stanley and Siever (2010) suggested that an altered regulation within the OT system might be an underlying mechanism for this interpersonal dysregulation in BPD, which might have developed because of or been enforced by adverse caretaking experiences in early childhood. Adverse early caretaking experiences, such as maltreatment, abuse, emotional neglect, separation, inconsistency, and invalidation, are thought to disrupt interpersonal attachment, leading to distinct attachment patterns (e.g., Lyons-Ruth and Jacobvitz, 2008). Current attachment representations of early experience can be assessed in adulthood by semi-structured interviews (Ravitz et al., 2010). These narrative assessments are typically used to divide individuals into groups with regard to representational security or insecurity, including individuals who are unresolved with regard to loss or physical or sexual abuse. More than 90% of adult BPD patients show insecure attachment classifications, and a high frequency of unresolved attachment representations predominates in some samples (Barone, 2003; Agrawal et al., 2004; Levy et al., 2006; Gunderson and Lyons-Ruth, 2008; Bakermans-Kranenburg and van IJzendoorn, 2009). Unresolved attachment, therefore, is of special importance in the development of BPD (Lyons-Ruth and Jacobvitz, 2008; Buchheim and George, 2011).

The neuropeptide OT has been hypothesized to play a crucial role in attachment, because it is involved in the regulation of human social behaviors such as mother-infant interaction, pair bonding, affiliative behavior, trust, and trustworthiness (Macdonald and Macdonald, 2010; Meyer-Lindenberg et al., 2011; Feldman, 2012). Intranasal administration of OT in healthy participants leads to enhanced theory of mind capacity, perception of attachment security, and relational trust and reduction of social stress (Kirsch et al., 2005; Kosfeld et al., 2005; Domes et al., 2007; Heinrichs and Domes, 2008; Buchheim et al., 2009; Zhong et al., 2012; Kumsta and Heinrichs, 2013). In BPD, OT dysregulation has been hypothesized to be one major neurobiological mechanism underlying interpersonal problems and hypersensitivity to social cues (Stanley and Siever, 2010; Meyer-Lindenberg et al., 2011). Intranasal OT was found to have stress-attenuating effects (reduced cortisol levels and reduced dysphoric mood) in 14 BPD patients during the TSST (Simeon et al., 2011). In neuroeconomic game paradigms, however, OT was found to exert different effects in BPD patients than in healthy participants, i.e., intranasal OT administration reduced trust and cooperation in 14 (four males) BPD patients and interpersonal trust in 13 (five males) BPD patients compared to HCs (Bartz et al., 2011; Ebert et al., 2013). These findings support the notion that sensitivity to social cues may be increased by OT, but the interpretation of these cues might be influenced by contextual (i.e., presence of a stranger versus friend) or individual (i.e., gender, attachment patterns, or the presence of psychiatric symptoms) factors (Olff et al., 2013). This might also be reflected in fewer prosocial behaviors in a study of non-verbal communicative signals in BDP during social interaction after OT administration (Brüne et al., 2015). However, another study showed that intranasal OT administration decreased the hypersensitivity to social threat in 40 female BPD patients, as measured by patterns of reflexive eye movements toward angry and fearful faces, and normalized amygdala activity (Bertsch et al., 2013). In addition, a recent study demonstrated that OT administration abolished the avoidant reaction to angry faces in 13 (five males) BPD (Brüne et al., 2013). Taken together, OT research is inconclusive and according to Brüne (2015) it seems oversimplified to render BPD an OT deficit syndrome (Stanley and Siever, 2010). Moreover it is still unclear whether OT should be used therapeutically as an add-on treatment in BPD patients (Eckstein and Hurlemann, 2013; Brüne, 2015). Inconsistent study results and effects of OT in BPD might be explained by inhomogenity and limitations of previous studies including mixed-sex and small size of samples as well as missing control for mestrual cycle in some cases.

Adverse attachment events during childhood, such as physical and sexual abuse by a significant caretaker, contribute to the development of BPD (Leichsenring et al., 2011), and maltreatment has been shown to be associated with altered OT regulation in children (Seltzer et al., 2014). The first study investigating OT plasma concentrations in 34 female BPD patients found lower plasma levels in patients than in HCs; levels were associated with childhood traumatization (Bertsch et al., 2012). A recent study by Jobst et al. (2014) showed lower OT plasma levels in 22 female BPD patients than in healthy individuals in response to a social exclusion situation, which constitutes a strong negative bonding stimulus in BPD (Jobst et al., 2014). These results converge with previous findings of an impaired social repair function in BPD when social cooperation is broken (King-Casas et al., 2008). The hypothesis of an altered regulation of endogenous OT in BPD is further supported by links to traumatic lifetime experiences: OT CSF levels of 22 women with childhood traumatization were negatively associated with trauma in a study categorizing participants into those with none-mild versus those with moderate-severe exposure to various forms of childhood abuse or neglect. The authors found inverse associations between CSF OT concentrations and both the number of exposure categories and the severity and duration of the abuse (Heim et al., 2009). Pierrehumbert et al. (2010) found a positive correlation between OT release after a social stress situation (TSST) and childhood trauma in a mixed-sex sample. The study included participants with sexual abuse in childhood or adolescence as well as survivors of cancer in childhood or adolescence and a control group. Participants with a life-threatening illness during childhood showed higher OT levels than both abused and healthy participants (Pierrehumbert et al., 2010).

Several studies have focused on the link between affiliative behavior, attachment, and OT (Feldman, 2012; Stoesz et al., 2013). Studies on the role of OT for bonding difficulties report evidence of an effect of OT on human affiliation, because they observed that OT plasma levels across pregnancy and the postpartum period predicted mother–infant bonding (Feldman et al., 2007; Eapen et al., 2014). Moreover, intranasal OT administration increased the subjective experience of attachment security in 26 healthy male participants with insecure attachment representations (Buchheim et al., 2009), and adult attachment representations predicted OT and cortisol response to stress in both individuals with (*n* = 46) and those without (*n* = 28) childhood trauma (Pierrehumbert et al., 2012). Participants with preoccupied attachment showed low OT levels after the stress situation, whereas participants with unresolved attachment showed intermediate OT levels (Pierrehumbert et al., 2012). A recent pilot study (*n* = 14) used film clips of bonding or abandonment scenes to investigate OT plasma levels as a potential biomarker for alterations in the female attachment system. While lower OT plasma levels during abandonment scenes were positively correlated with posttraumatic stress symptoms, higher OT plasma levels during bonding scenes were negatively correlated with scores of dissociation and somatization (Munro et al., 2013).

To our knowledge, there is no study published investigating the association between attachment patterns in BPD patients and OT plasma concentrations. As the first analysis of our study was mainly focusing on differences in changes of peripheral OT levels after social exclusion between BPD patients and HCs (Jobst et al., 2014) irrespective of attachment representations, we re-analyzed our data here including the results of the AAP, a reliable and valid measure of adult attachment representations (George et al., 1999; George and West, 2011, 2012). We investigated attachment patterns in relation to both, general OT levels and their change during a social exclusion situation comparing BPD patients and HC. We hypothesized that alterations of OT levels observed in BPD patients would be especially pronounced in patients classified as unresolved, because this group is most frequently and seriously affected by early attachment trauma (Stalker and Davies, 1995; Bakermans-Kranenburg and van IJzendoorn, 2009) potentially disrupting a physiological development of OT regulation.

## Materials and Methods

### Participants

Twenty-two female patients aged 19 to 46 years (*M* = 30.0 years, *SD* = 7.95) diagnosed with BPD and 21 HC aged 19 to 50 years (*M* = 29.71, *SD* = 10.26) matched for gender, age, and education participated in this study. BPD patients (8 inpatients and 14 outpatients) were recruited at the Department of Psychiatry and Psychotherapy at the Ludwig Maximilian University, Munich. BPD diagnoses and comorbid axis I and II diagnoses were assessed with the German Version of the SCID (SCID-I screening, SCID-II interview; First et al., 1995). Exclusion criteria were a comorbid diagnosis of substance dependence, schizophrenia, schizoaffective disorder, or bipolar disorder. All BPD patients met diagnostic criteria for BPD according to DSM-IV criteria. Comorbid diagnoses on SCID-I and -II were *M* = 3.50, *SD* = 1.68 (SCID-I: *M* = 0.91, *SD* = 0.81; range 0–3; SCID-II: *M* = 2.59, *SD* = 1.44, range 0–5). The following comorbid personality disorders were observed: Avoidant (*n* = 11), depressive (*n* = 10), anankastic (*n* = 4), negativistic (*n* = 4), paranoid (*n* = 4), dependent (*n* = 2), and histrionic (*n* = 1). Other comorbidities were major depressive disorder (*n* = 5) and eating disorder (*n* = 3). HC were recruited by advertisements in newspapers and posters on noticeboards. In HC, exclusion criteria were a current psychiatric disorder and a psychiatric and psychological treatment in the past 10 years, as confirmed with the SCID-I and -II screening instruments (First et al., 1995).

Most BPD patients received pharmacological treatment, as follows: Antidepressants (*n* = 15), SGA (*n* = 14), and mood stabilizers (*n* = 8). At the time of assessment, seven patients were taking sedative medication, and three patients were not taking any psychopharmacological drugs. All BPD patients were in current inpatient or outpatient psychotherapy.

The study was approved by the Institutional Review Board of the Faculty of Medicine at the University of Munich. All participants provided written informed consent. The study was part of a larger study investigating changes of neuropeptides during a social exclusion paradigm in patients with different psychiatric diagnoses. The results presented here are an extension of our previous study (Jobst et al., 2014).

### Procedure and Measures

The experimental protocol included three separate sessions. At the first session, a screening interview was conducted to check for exclusion criteria, and participants were asked to provide informed consent. Moreover, participants completed German versions of a series of psychometric questionnaires: The BSL-23 (Bohus et al., 2009) to measure the severity of BPD symptoms; the BDI-II (Beck et al., 1996), and the 24-item HAMD-24 (Hamilton, 1960) to measure the severity of depression; the emotional abuse, emotional neglect, physical abuse, physical neglect, and sexual abuse subscales of the CTQ (Bernstein et al., 2003; Klinitzke et al., 2012), to assess traumatic experiences in childhood; and the RSQ (Downey and Feldman, 1996) to check for the grade of sensitivity to rejection.

At the second session, trained professionals at the Department of Psychiatry and Psychotherapy, Ludwig Maximilian University, Munich, administered the AAP (George et al., 1999; George and West, 2011, 2012). Administration lasted about 30 min. The AAP is a free-response measure comprising a set of eight line drawing stimuli, one showing a neutral scene and seven showing attachment scenes (e.g., illness, separation, solitude, death, and threat). Participants are asked to tell a story of what led up to a scene, what the characters are thinking or feeling, and what might happen next. Classifications are derived from evaluating response patterns on several response dimensions (agency of self, connectedness, synchrony, defensive processes) by using verbatim transcripts of audiotaped responses to the seven attachment-activating stimuli. The coding was performed by independent, trained raters at the Department of Psychology at the University of Innsbruck, Austria. Each participant was assigned to one of the four attachment classification groups “secure,” “dismissing,” “preoccupied,” or “unresolved.” The last three groups represent different types of attachment insecurity. Among these, unresolved representation is most frequently associated with early attachment trauma (Stalker and Davies, 1995) and is assigned when the response material demonstrates a failure to organize or contain representation indications of fear and dysregulation. On the basis of this differentiation, we defined two categorical pairs of representation types for comparison: (1) Secure vs. insecure, and (2) organized (resolved) vs. unresolved (disorganized). The psychometric properties of the AAP are excellent (George and West, 2012). Interrater reliability was found to be 90% (*k* = 0.85, *p* < 0.001, *n* = 144). Test–retest reliability was calculated on the basis of 69 participants who completed the AAP retest 3 months after the original AAP administration; 58 (84%) were classified in the same attachment group categories (*k* = 0.78, *p* < 0.001; 82% stability for secure, 96% stability for dismissing, 62% for preoccupied; 80% for unresolved). Verbal intelligence and social desirability are not related to AAP classifications (George and West, 2012). AAP convergence of classifications with the AAI (George et al., 1985; Main and Goldwyn, Main and Goldwyn), the gold standard in the field of adult attachment assessment, was 84% for the four-group classification (*k* = 0.71, *p* < 0.001), 91% for the two-group classification (*k* = 0.91, *p* < 0.001), and 88% for the unresolved-resolved classification (*k* = 0.75, *p* < 0.001). The predictive validity of the AAP has been established for both clinical and healthy samples (Buchheim and George, 2011, 2012; George and West, 2012).

At the third session, plasma samples were taken for OT measurement before and after individuals participated in a social exclusion paradigm (Cyberball) in a standardized experimental setting, as previously reported (Jobst et al., 2014). In brief, through-the-wall blood drawings were performed by using a long catheter that ran through a soundproof lock to an adjacent laboratory. Participants had no visual contact with the investigators during the experiment. After baseline measurements had been taken, the Cyberball game was started on a computer screen positioned in front of the participants. The Cyberball paradigm is a virtual ball-tossing game played with two other virtual players and controlled by computer software (Williams and Jarvis, 2006). Participants were shown a statement on the computer screen that told them that they would take part in this virtual game with two other real players. Participants played one round of Cyberball, which lasted about three minutes. Participants were thrown the ball three times (10% of the throws) at the beginning of the round but were then excluded from the game without explanation and were able only to watch the other players.

During our study, we paid special attention to the stage of participants’ menstrual cycle, because of neuroendocrine interactions with OT. Participants taking hormonal contraception were assessed during the 3rd and 18th day of the intake period, and those not taking hormonal contraception were assessed within the follicular phase, between the 5th and 12th day of the menstrual cycle, because gonadal hormones are more stable during this period (Salonia et al., 2005). Participants were told not to eat or drink one hour before hormonal measurement. All measurements took place in the morning between 8 and 11 a.m. to control for the circadian change in hormones.

Blood samples for OT measurement were collected at four time points: t0 (baseline), t1 (5 min after Cyberball), t2 (15 min after Cyberball), and t3 (40 min after Cyberball). Storage tubes were prepared in advance with aprotinin (500 IU/ml) in order to prevent OT degradation. Afterward, blood samples were centrifuged (1600*g*, 15 min), and plasma was stored at minus 80°C until the biochemical analyses. Analysis of plasma OT was performed by the Neurochemical Laboratory at the Department of Psychiatry, Ludwig Maximilian University, Munich, with a commercially available Enzyme Linked Immunosorbent Assay (ELISA) Kit (Enzo Life Sciences, Germany). Measurements were performed in duplicates. The inter-assay CV was below 17%, and the intra-assay CV was 15%. To control for stress reactivity, plasma cortisol was also measured; serum cortisol levels were determined with an immunoassay analyzer (Elecsys Cortisol Test; Roche Diagnostics, Mannheim, Germany), according to the manufacturer’s instructions.

Emotional reactions to the social exclusion paradigm were measured with self-rating questionnaires, the NTQ (Williams et al., 2000), the Emotion Scale (Gross and Levenson, 1995) before and after Cyberball, as previously reported (Jobst et al., 2014). In addition, we asked the participants to assess aversive inner tension, expressed as a percentage of maximal tension before and after Cyberball.

### Statistics

SPSS software version 17 was used for statistical analyses. Demographic and psychometric analyses were performed with χ^2^-tests and independent sample *t*-tests. To analyze outcomes in the Cyberball paradigm, we used paired sample *t*-tests for the NTQ and rmANOVA for the emotion scale. For analysis of OT and cortisol levels as well as their changes during Cyberball, three repeated measure analyses of variance (rmANOVA) were conducted: (1) A group (HC vs. BPD) × time (before vs. after) rmANOVA (as reported in Jobst et al., 2014); (2) in BPD patients only a group (unresolved vs. other) × time (before vs. after) rmANOVA; and (3) in HC only a group (secure vs. insecure) × time (before vs. after) rmANOVA. In case of violation of sphericity assumption, Huynh–Feldt correction was used to present data. Moreover, we applied *post hoc* independent sample *t*-tests. Baseline OT plasma measurements showed a wide interindividual range of 164.4 pg/ml to 5092.4 pg/ml. Two BPD patients and two HC were excluded from statistical analysis, because their OT levels were higher than three standard deviations from the mean of the whole sample (outliers between 1271.2 and 5092.4 pg/ml). Regarding attachment representations, data for 19 BPD patients and 18 HC were available and included into further statistical analyses. Data samples for baseline OT showed normal distribution (Kolmogorov–Smirnov test). Correlation analyses were performed by Pearson correlation.

## Results

### Demographic and Clinical Characteristics

Borderline personality disorder patients and HC did not differ significantly with regard to age and education. As expected, BPD patients showed significantly higher scores than HC for borderline and depressive symptoms (BSL-23, HAMD, and BDI-II). Also as expected, the BPD group showed higher CTQ scores, indicating early childhood trauma. On the basis of CTQ cut-off values defined by Bernstein and Fink (1998), the BPD patients in our sample reported moderate to severe traumatization. The CTQ scores of the control group were below cut-off values on all subscales. BPD patients also showed significantly higher scores of rejection sensitivity than controls, as assessed by the RSQ. There was no significant difference between BPD and HC as regards menstrual cycle and hormonal contraception. For an overview see **Table 1**.

**Table 1:** Sample characteristics of patients with BPD and HC.

Characteristics	BPD patients *(n* = 20)	HC *(n* = 19)	*t*-test	*p*-value
	Mean *(SD)*^f^			
Age (in years)	29.85 (7.46)	30.42 (10.55)	0.20	0.847
Education (in years)	11.20 (1.70)	12.0 (1.53)	1.83	0.132
BSL-23^a^	2.07 (0.96)	0.08 (0.13)	-9.18	<0.001
BDI-II^b^	32.75 (14.28)	2.33 (3.0)	-9.30	<0.001
HAMD-24^c^	29.90 (12.04)	1.28 (1.57)	-10.20	<0.001
CTQ^d^ Emotional abuse Physical abuse Sexual abuse Emotional neglect Physical neglect Global	17.89 (4.98) 10.80 (7.16) 12.35 (8.39) 18.30 (5.19) 11.20 (4.4) 15.00 (4.86)	8.22 (6.26) 5.56 (1.34) 5.44 (2.20) 7.94 (3.95) 6.61 (2.59) 6.76 (2.35)	-3.19 -3.05 -3.39 -6.86 -3.96 -6.76	0.003 0.004 0.002 <0.001 <0.001 <0.001
RSQ^e^	17.92 (5.45)	5.98 (2.82)	-8.47	<0.001
Day of menstrual cycle Hormonal contraception	9.83 (4.15) 5 (22.7%)	8.78 (3.62) 10 (47.6%)	-0.813 X^2^: 2.931	0.422 0.091

*No significant differences between patient and control groups in age and education. ^a^BSL-23, Borderline Symptom List; ^b^BDI-II, Beck Depression Inventory; ^c^HAMD-24, Hamilton Depression Rating Scale; ^d^CTQ, Childhood Trauma Questionnaire; ^e^RSQ, Rejection Sensitivity Questionnaire; ^f^SD, standard deviation.*

#### Attachment, Clinical Variables, and Trauma

None of the BPD patients showed a secure attachment representation, whereas 8 HC (44.4%) were classified as secure. The attachment representations in the BPD group were as follows: 12 (63.2%) unresolved (disorganized), 4 (21.1%) dismissing, and 3 (15.8%) preoccupied, making a total of 36.8% with organized representation (dismissing and preoccupied together). Attachment representations among the HC were as follows: 8 (44.4 %) secure, 6 (33.3%) dismissing, 4 (22.2%) preoccupied, and none unresolved (disorganized). Secure and unresolved (disorganized) attachment representation differed significantly between BPD and HC (*secure*: χ^2^ = 10.77; df = 1; *p <*0.001*; unresolved:* χ^2^ = 16.83; df = 1; *p <*0.001), whereas dismissing and preoccupied representations did not (*dismissing*: χ^2^ = 0.71; df = 1; *p* = 0.407; *preoccupied*: χ^2^ = 0.25; df = 1; *p* = 0.622).

The group of organized and unresolved (disorganized) BPS patients showed no significant difference with regard to age, education, menstrual cycle, contraception, borderline symptoms (BSL-23), depressive symptoms (HAMD, BDI-II), and rejection sensitivity (RSQ) (overview **Table 2**). According to CTQ cut-off values, unresolved (disorganized) BPD patients reported severe traumatization on all trauma subscales, whereas organized BPD patients reported severe traumatization only on the subscales emotional abuse, emotional neglect, and physical neglect and mild traumatization on the subscales physical abuse and sexual abuse. The differences in the last two subscales were significant: On the physical abuse subscale, *n* = 5 (58.3%) patients in the unresolved (disorganized) group showed severe traumatization (cut-off score > 10) versus *n* = 2 (8.3%) in the organized group (χ^2^ = 10.67; df = 1; *p <*0.001). On the sexual abuse subscale, *n* = 3 (12.5%) patients in the unresolved (disorganized) group showed severe traumatization (cut-off score > 8) vs. *n* = 8 (66.7%) in the organized group (χ^2^ = 11.06; df = 1; *p <*0.001). HC with secure and insecure attachment representations did not differ with regard to age or education. However, insecure HC scored significantly higher on the HAMD (*t* = 2.649; df = 11.227; *p* = 0.022) than secure HC.

**Table 2:** Sample characteristics of BPD patients with organized or disorganized attachment representation.

Characteristics	Organized (*n* = 7)	Unresolved (*n* = 12)	*t*-test	*p*-value
	Mean (*SD*)^f^			
Age (in years)	25.14 (4.85)	31.75 (7.49)	-2.08	0.053
Education (in years)	12.0 (1.73)	10.83 (1.64)	1.47	0.161
BSL-23^a^	1.74 (1.09)	2.05 (1.11)	-0.60	0.558
BDI-II^b^	30.86 (16.82)	33.5 (13.91)	-0.371	0.716
HAMD-24^c^	27.86 (9.37)	30.42 (13.87)	-0.432	0.671
CTQ^d^ Emotional abuse Physical abuse Sexual abuse Emotional neglect Physical neglect Global	16.0 (6.46) 8.57 (7.39) 8.43 (7.46) 16.86 (6.01) 10.43 (4.28) 12.06 (5.61)	19.0 (3.77) 12.58 (7.01) 14.75 (8.66) 18.58 (4.62) 11.17 (4.49) 15.22 (4.43)	-1.29 -1.18 -1.61 -0.70 -0.35 -1.36	0.214 0.253 0.126 0.491 0.730 0.191
RSQ^e^	17.06 (4.46)	18.03 (6.23)	-0.36	0.727
Day of menstrual cycle Hormonal contraception	8.33 (2.81) 3 (42.4%)	10.6 (5.04) 1 (9.1%)	-1.064 X^2^: 3.273	0.333 0.195

*^a^BSL-23, Borderline Symptom List; ^b^BDI-II, Beck Depression Inventory; ^c^HAMD-24, Hamilton Depression Rating Scale; ^d^CTQ, Childhood Trauma Questionnaire; ^e^RSQ, Rejection Sensitivity Questionnaire; ^f^SD, standard deviation.*

#### OT and Cortisol Plasma Levels

Though plasma OT levels showed a marked inter-individual variation, they were remarkably stable at an individual level.

##### Comparison of OT levels in BPD vs. HC

**FIGURE 1:**
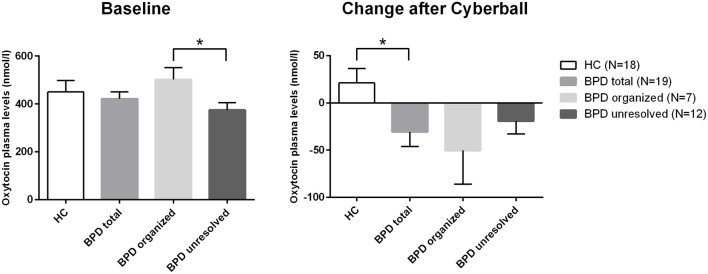
**Differences in baseline plasma OT levels and differences in the changes of plasma levels of OT over time (before and after Cyberball).** *significant at α = 0.05.

The rmANOVA comparing BPD patients and HC revealed a significant group (BPD vs. HC) × time (before vs. after) interaction when conducted over the first two time points (*F* = 4.957; df = 1; *p* = 0.032; **Figure 1**) and no significant group × time interaction when conducted over the four time points (*F* = 1.467; df = 2.427; *p* = 0.234): OT levels dropped after Cyberball from t0 to t1 in BPD whereas levels increased in HC (*post hoc* independent sample *t*-test: *t* = 2.227; df = 37; *p* = 0.032), as previously reported (Jobst et al., 2014).

##### Comparison of OT levels in BPD patients with unresolved vs. organized attachment patterns

As there were neither BPD patients with secure nor HCs with unresolved attachment, unresolved vs. organized attachment patterns were compared in BPD patients only and insecure vs. secure attachment patterns in HC only. For BPD patients, the respective rmANOVA showed a trend toward a significant group effect between BPD patients with unresolved (disorganized) and organized attachment (*F* = 3.679; df = 1; *p* = 0.073). BPD patients with unresolved (disorganized) attachment representations tended to show constantly lower OT levels compared to HC. *Post hoc* independent sample *t*-test showed significantly lower OT plasma concentrations at baseline in BPD patients with unresolved (disorganized) attachment than in BPD patients with an organized attachment representation (*t* = 2.346; df = 17; *p* = 0.031, *d* = –1.078; see **Figure 1**). There was no significant effect of time (before vs. after) in BPD (*F* = 1.647; df = 3; *p* = 0.201), nor a significant group (unresolved vs. organized) × time (before vs. after) interaction (*F* = 0.361; df = 2.453; *p* = 0.742); both groups showed a similar course of OT levels after social exclusion (**Figure 1**).

##### Comparison of OT levels in HC with insecure vs. secure attachment patterns

The respective rmANOVA for HC showed neither a significant effect of group (secure vs. insecure attachment pattern: *F* = 0.012; df = 1; *p* = 0.916) nor of time (before vs. after: *F* = 0.587; df = 3; *p* = 0.627) and no significant group × time interaction (*F* = 0.450; df = 3; *p* = 0.718).

##### Comparison of cortisol levels between groups

Cortisol plasma levels were analyzed using rmANOVAs as described above. There were no significant effects of group or group × time interaction between BPD and HC, BPD patients with organized and unresolved (disorganized) attachment representation, and HC with secure and insecure attachment representation, respectively. There was a significant effect of time: Cortisol levels significantly decreased in BPD and HC (Jobst et al., 2014) and this decrease was not influenced by attachment representation. OT and cortisol plasma levels for all groups and time points are shown in **Table 3**.

**Table 3:** Oxytocin plasma levels in patients with BPD and HC according to attachment representation.

	BPD (*n* = 19)	HC (*n* = 18)	BPD		HC	
			Organized (*n* = 7)	Unresolved (*n* = 12)	Secure (*n* = 8)	Insecure (*n* = 10)
	
	Mean (*SD*)					
Oxytocin t1	421.67 (127.70)	450.26 (204.42)	502.14 (130.71)	374.73 (104.11)	443.21 (195.30)	455.89 (221.79)
Oxytocin t2	390.97 (130.70)	471.46 (240.63)	451.73 (186.02)	355.53 (73.21)	451.46 (225.54)	487.56 (263.01)
Oxytocin t3	399.19 (142.60)	468.19 (252.68)	480.60 (175.38)	351.70 (99.09)	466.55 (237.54)	469.50 (276.96)
Oxytocin t4	403.58 (128.54)	454.22 (233.85)	468.36 (133.92)	362.36 (111.91)	456.21 (214.54)	452.62 (259.80)
Cortisol t1	530.04 (236.08)	505.06 (170.52)	635.37 (298.52)	468.60 (188.24)	468.65 (208.98)	454.19 (120.35)
Cortisol t2	522.67 (243.70)	523.87 (185.45)	632.50 (299.91)	458.61 (170.06)	589.81 (223.08)	471.11 (138.41)
Cortisol t3	499.47 (246.31)	497.53 (195.64)	631.53 (312.10)	422.44 (169.03)	563.89 (244.15)	444.44 (137.44)
Cortisol t4	446.24 (221.78)	448.16 (198.26)	554.80 (286.38)	377.16 (144.61)	519.78 (239.67)	390.87 (146.30)

### Psychological Measures during Cyberball

The results of the psychological measures during Cyberball in BPD patients and HC have been published elsewhere (Jobst et al., 2014): To summarize, BPD patients felt more readily excluded, and the exclusion paradigm had a stronger negative impact on their needs than it did in HC (Needs-Threat-Questionnaire), but the social exclusion paradigm did not have impact on inner tension, neither in BPD, nor in HC. Negative emotions focused on other significantly increased in BPD (rmANOVA: time effect: *F* = 6.065; df = 1; *p* = 0.019; group effect: *F* = 14.474; df = 1; *p* = 0.001; group × time interaction: *F* = 2.603; df = 1; *p* = 0.115). *Post hoc t*-tests revealed that especially anger and contempt significantly increased in BPD (anger *t* = –2.251; df = 1; *p* = 0.037; contempt: *t* = 2.480; df = 18; *p* = 0.023), which was not the case in HC (Jobst et al., 2014).

We conducted additional analyses independently for BPD and HC regarding the association between attachment representation and psychological reactions toward social exclusion. BPD with organized and disorganized attachment representations felt disregarded (*t* = 0.903; df = 15; *p* = 0.381) and excluded (*t* = 2.095; df = 11.311; *p* = 0.059) to a comparable degree and judged ball ownership similarly (*t* = 0.445; df = 15; *p* = 0.663) and realistically (organized: 11.37%; disorganized: 9.85%). Moreover there was no significant difference between organized and disorganized BPD regarding the aversive impact triggered by the social exclusion (NTQ aversive impact factor: *t* = 0.736; df = 14; *p* = 0.474). Negative emotions focused on others increased in both the organized and disorganized group (rmANOVA: time effect: *F* = 4.385; df = 1; *p* = 0.053), but did not show differences between groups (group effect: *F* = 0.128 df = 1; *p* = 0.725) nor a significant group × time interaction (*F* = 0.015; df = 1; *p* = 0.905). However, contempt significantly increased in the organized group whereas this was not the case for the disorganized group (rmANOVA: time effect: *F* = 9.120; df = 1; *p* = 0.008; group effect: *F* = 0.337; df = 1; *p* = 0.569; group × time effect: *F* = 3.446; df = 1; *p* = 0.082). There was no such difference for anger. Inner tension did not differ between groups. Analysis between secure and insecure attached HC revealed no significant different emotional response to social exclusion: Both groups felt disregarded (*t* = 1.065; df = 16; *p* = 0.303) and excluded (*t* = 1.056; df = 16; *p* = 0.306) to a comparable degree and judged ball ownership similar (*t* = –0.654; df = 16; *p* = 0.523) and realistically (secure: 8%; insecure 10%). Both groups demonstrated a similarly aversive impact factor (*t* = 0.565; df = 16; *p* = 0.580). Change of other focused negative emotions and inner tension did not differ between groups.

## Discussion

Insecure and especially unresolved attachment representations are predominant patterns in patients with BPD. In the present study, we were able to replicate this finding on a behavioral level. Our main finding, however was, that BPD patients with unresolved attachment show significantly lower baseline OT plasma levels than BPD patients with organized attachment. These results extend previous findings of lower plasma OT baseline levels in BPD than in HC and of a negative association between OT plasma levels and childhood trauma (Bertsch et al., 2012). The lower OT plasma levels in unresolved attached BPD seem to be plausible when taking into account that unresolved attachment trauma is associated with severe childhood trauma (Stalker and Davies, 1995; Bakermans-Kranenburg and van IJzendoorn, 2009). Accordingly, in our study BPD patients with unresolved attachment representations showed significantly more severe physical and sexual childhood abuse compared to BPD individuals with organized attachment. In our small sample, we could not find a direct association between childhood trauma (CTQ) and OT plasma levels, however, smaller OT recovery was associated with higher physical and emotional abuse (Jobst et al., 2014). Therefore it would be of major interest to investigate in future studies the association between childhood trauma, unresolved attachment representation, and OT in larger samples of BPD patients.

Oxytocin change after social exclusion (Cyberball) – a relevant social stimulus (Williams and Jarvis, 2006; Williams, 2007) that activates social pain in humans (Eisenberger et al., 2003) – did not differ in regard to attachment representations (not in BPD, nor in HC group). Both BPD patients with organized and those with unresolved attachment representations showed similar courses of OT after social exclusion; though we found lower OT plasma levels in BPD patients with unresolved attachment representations at every time point. Comparing BPD and HC group, change of OT plasma levels directly after social exclusion significantly differed as previously described (Jobst et al., 2014): After social exclusion OT plasma levels tended to decrease in BPD patients and tended to increase in HC and this different course was significant. Descriptively OT plasma levels were constantly lower in BPD patients than in HC. In accordance with other studies (Zöller et al., 2010; Zwolinski, 2012), the social exclusion paradigm did not elicit a neurobiological stress reaction, as central stress pathways might be inhibited due to the presence of a defensive response (emotional analgesia) (Bass et al., 2014).

Emotional responses to social exclusion differed between BPD and HC as previously described (Jobst et al., 2014). Attachment representation did not modify these responses besides a significant higher increase of contempt in the organized vs disorganized BPD group. We hypothesize that OT release after social exclusion might be understood as neuroendocrine reaction to broken relations. In this context, OT release may reduce social pain and induce pro-social orientation. In BPD patients, this OT reaction to social exclusion may be impaired, possibly corresponding to the general difficulty of BPD patients in repairing broken cooperation (King-Casas et al., 2008).

In previous studies, BPD patients responded to intranasal OT administration in the opposite way to HC, i.e., intranasal OT reduced trust in BPD but increased trust in HC (Bartz et al., 2011). Moreover, in depressed patients OT administration in addition to psychotherapy even increased fear during psychotherapy (Macdonald et al., 2013). Taking these results into consideration, another hypothesis seems plausible. Reduced OT levels in BPD as found in our study, and especially in BPD patients with unresolved attachment representations, might be the result of an altered OT system, most likely resulting from early caretaking experiences (Meaney, 2001; Champagne, 2008; Bertsch et al., 2013; Herpertz and Bertsch, 2015), so that the patients have a baseline state of “low OT.” Lower OT levels in BPD compared to HCs might be specifically found in BPD with unresolved attachment representations but not in BPD with organized ones. Consequently, this “low OT state” might represent a protective state in which social pain might be more bearable, with reduced perception of negative emotions. OT administration might take the patients out of this state and consequently increase fear. Experiences occurring in sensitive periods in early development, especially traumatic childhood experiences, might critically modify epigenetic states of genes and influence the developing OT system and according to Kumsta and Heinrichs (2013) epigenetic modification of genes involved in OT signaling might therefore be involved in the mechanisms mediating the long-term influence of early adverse experiences on socio-behavioral outcomes. In this context the development of BPD might be understood as a result of early childhood maltreatment with the development of insecure attachment representations, which then contribute to Borderline symptoms in adulthood. This relationship might be neurobiologically mediated by epigenetic changes, which can be pictured by changes within the OT system. However, Brüne (2015) suggests that in the spectrum of childhood trauma, emotional neglect in early childhood may have a different impact on the OT system than, e.g., sexual abuse or later traumatic experiences in adolescence.

Our study has some limitations. These include a small sample size, pharmacological treatment in the BPD group, especially intake of neuroleptic medication, and failure to measure estrogen and progesterone levels. Due to the small sample size and exploratory character of this study, we did not control for influencing factors such as medication or depression. This should be re-analyzed within a larger sample. Moreover, the specificity of a dysregulated OT system to BPD patients is still unclear, as well as the interaction of OT with other neuromodulators, such as the endocannabinoid system, which has been found relevant for pain processing (Herpertz and Bertsch, 2015). The meaning and reliability of OT plasma levels with regard to central nervous system processes is still a matter of debate. Recently published articles argue that peripheral OT cannot give information about cognition, emotion and behavior (Meyer-Lindenberg et al., 2011; Quintana and Woolley, 2015; Leng and Ludwig, 2016; Walum et al., 2016). However, several studies also state the opposite and demonstrate an association between peripheral and central OT measures (Dal Monte et al., 2014; Carson et al., 2015; Freeman et al., 2016). Future research should clarify this controversy. Therefore our results have to be interpreted with caution. Another bias might result from the analysis of OT levels in a non-extracted plasma sample using a commercially available ELISA, which limits a direct comparison with other studies that used extracted samples and radioimmunoassays (Szeto et al., 2011).

In summary, this study demonstrates that BPD patients with unresolved attachment representations have significantly lower baseline OT plasma levels than BPD patients with organized attachment representations. However, this finding needs to be replicated in a larger sample.

## Author Contributions

Organized and designed the whole study setting and experiments: AJ, FP, PF, BR, AB. Coded, analyzed, and controlled the data and wrote most parts of the manuscript: AJ, FP, MG, AB. Performed and analyzed the experiments and data: MM, TD, CB-S, LS, NS, PZ.

## Conflict of Interest Statement

The authors declare that the research was conducted in the absence of any commercial or financial relationships that could be construed as a potential conflict of interest.

## Abbreviations

AAI: Adult Attachment Interview
AAP: Adult Attachment Projective Picture System
BDI-II: Beck Depression Inventory
BPD: Borderline personality disorder
BSL-23: Borderline-Symptom-List-23
CSF: cerebrospinal fluid
CTQ: Childhood Trauma Questionnaire
CV: interassay coefficient of variation
HAMD-24: Hamilton Depression Rating Scale
HC: healthy controls
NTQ: Needs-Threat-Questionnaire
OT: Oxytocin
RSQ: Rejection Sensitivity Questionnaire
SCID: Structural Clinical Interview for DSM-IV
SGA: second-generation antipsychotics
TSST: Trier Social Stress Test
